# The Social Functionality of Humor in Group-Based Research

**DOI:** 10.1177/1049732318800675

**Published:** 2018-10-19

**Authors:** Rebecca Hewer, Katherine Smith, Gillian Fergie

**Affiliations:** 1 University of Edinburgh, Edinburgh, Scotland; 2 University of Glasgow, Glasgow, Scotland

**Keywords:** humor, citizens’ juries, deliberative democracy, power, reflexivity, qualitative, United Kingdom

## Abstract

Citizens’ juries provide deliberative fora within which members of the public can debate complex policy issues. In this article, we reflect on our experience of undertaking three citizens’ juries addressing health inequalities, to explore the positive and facilitative role that humor can play within group-based research focusing on sensitive health policy issues. We demonstrate how both participants and researchers engaged in the production of humor in ways which troubled prevailing power dynamics and facilitated positive relationships. We conclude by recommending that researchers, particularly health policy researchers and those pursuing the kind of lengthy group-based fora associated with deliberative research, consider the positive role humor can play when engaged reflexively. In so doing, we make a major contribution to extant literature on both deliberative fora (which is yet to consider humor’s facilitative capacities) and the role of humor in qualitative (health) research (which rarely explores researcher complicity in humor production).

## Introduction

In July 2016, we undertook three citizen juries (in Glasgow, Manchester, and Liverpool) to explore how a diverse range of lay participants perceived health inequalities. For the purposes of this article, citizens’ juries are group-based research encounters, during which members of the public consider evidence regarding politically salient problems and deliberate over possible policy responses. In utilizing this methodology, our primary intent was to explore substantive questions regarding public understandings of long-standing health asymmetries and potential policy responses. On analyzing our data, however, we were prompted to reflect on our methodological approaches, assumptions, and skills. This article employs some consequent (inductively generated) findings to explore the social functionality of humor within prolonged (in our case, 2-day) group-based research encounters, particularly with regard to the negotiation of power.

In what follows, we highlight multiple instances of humor as a dynamic arising from, and functioning within, our citizens’ juries—paying particular regard to its role in the negotiation of power-inflected research relationships. Furthermore, and where apposite,^1^ we seek to illuminate such instances by exploring how relatively ingrained social positionalities (e.g., gender) came to bear on research practices. In contrast to the existing literature, we reflect on how participants *and researchers* ethically deployed humor to negotiate power dynamics. We conclude by suggesting that humor *can be* an appropriate and potentially productive “facilitative [research] tool” (Browne, 2016, p. 201), which assists in the negotiation of research relationships, troubles traditional distributions of power, and facilitates potentially fraught discussions. In addition, we posit that humor may be a particularly powerful tool for qualitative health policy research, insofar as it enables participants to aptly navigate the relationships, power asymmetries, and substantive topics often explored within this domain. Whether humor realizes this potential, we suggest, depends on its reflexive and critical deployment, as well as the social capacities of participants.

We begin here by briefly describing citizens’ juries as a research method. We then explore humor as an object of academic interest: highlighting both explanatory theories and work which address humor as a practice. We also briefly discuss how humor has been broached in extant health research. Thereafter, we offer a concise and critical operationalization of power to make clear how we intend to employ this key concept throughout the article. Thereafter, we outline our methodological approach, before discussing our findings as they relate to the social utility of humor in group-based health policy research.

## Citizens’ Juries

The citizen’s jury—populated by a small group of demographically diverse individuals (the “jurors”)—provides a space within which members of the public can contemplate and discuss complex policy issues (Wakeford, 2002). They are structured encounters, which routinely involve preconceived activities designed to help participants consider evidence and debate potentially desirable policy approaches. Juries are intended, in essence, to be deliberative spaces, apt to challenge prevailing organizations of power by legitimizing knowledge generated by a nonspecialist citizenry. In some cases, they facilitate public engagement in democratic processes (Carney & Harris, 2013), but in many others, as in ours, they are used for research purposes, albeit with some effort to bring findings to the attention of policy audiences (Street, Duszynski, Krawczyk, & Braunack-Mayer, 2014). In our case, the aim of our juries was to gain a better understanding of public views about health inequalities and potential policy responses.

Citizens’ juries are group-based research encounters which resemble focus groups, but which diverge in a number of important respects. In short, while focus groups tend to be relatively brief endeavors, citizen juries can last for days, often requiring the development of more sustainable research relationships. Furthermore, while focus groups may demand minimal facilitator intervention, citizens’ juries often require a more directive approach: Juries tend to be quite heavily structured, and jurors are guided through a variety of research stages, including evidence consumption, deliberation, and identification of preferred policies. This can, in turn, induce distinct power dynamics.

Citizens’ juries are an innovative but increasingly popular method of data collection, particularly within health research. A 2014 systematic review of the use of citizens’ juries in health policy research identified 37 studies that, between them, reported results from 66 juries (Street et al., 2014). Beyond this, recent examples of health policy debates in which citizens’ juries have played an important role include the Irish Referendum on abortion and debates about tackling obesity in the Australian state of Victoria (Wise, 2017)

## The Constitution and Social Functionality of Humor

Multiple theoretical narratives strive to explain humor’s fundamental constitution and purpose. In the main, academic explorations of humor engage with three theories: superiority, incongruity, and relief (Lippitt, 1994; Meyer, 2000; Morreall, 2014; Sen, 2012; Watson, 2015). Superiority theory suggests that humor arises from pleasurable feelings of superiority toward another or our past selves (Sen, 2012; Watson, 2015). Its outward manifestations—jokes and laughter—deride and diminish those to whom they are oriented. According to this theory, humor is necessarily contemptuous and derisive. Superiority theory has been criticized for eliding humor in its entirety with its subset (e.g., ridicule) and for consequently promoting a partial account (Watson, 2015). Furthermore, critics highlight numerous situations in which superiority may be felt but not enjoyed (e.g., when encountering a stigmatized “other”), suggesting that the theory does little to illuminate humor in its breadth *or* specificity (Morreall, 2014).

Incongruity theory—perhaps the most academically popular (Watson, 2015)—claims that humor arises from a recognition of that which is incongruous and therefore unexpected (Lippitt, 1994; Sen, 2012; Watson, 2015). Humor is understood to result from a kind of violation of “natural” logic—narratives which deviate from their expected linearity, punch lines which subvert apprehension (Lippitt, 1994). In contrast to critiques of superiority theory, incongruity theory has been criticized for its unsustainable breadth. Not all incongruities are comic—some are even tragic (Morreall, 1989). A parent who does not love their child might be considered culturally incongruous, but does not necessarily invite hilarity. Furthermore, and as Lippitt (1994) argues, the necessary contingency of what is deemed “incongruous” demands we entertain an ongoing consideration of context—that which is humorous to one person might prove inconsequential to another.

In contrast to its counterparts, relief theory, which originates from Freudian analytics, focuses less on why we find events humorous and more on the physiological effects of laughter. Proponents posit that laughter releases tension, fear, and stress and is, therefore, a discharge of internal energy (Lippitt, 1994; Sen, 2012). Some theorists interpret Kantian conceptions of humor as bridging a divide between relief theory and incongruity theory, insofar as he posits that laughter is an expression of disappointed expectation, a release of tension when the anticipation of congruity is met with its opposite (Watson, 2015). Relatedly, much health literature suggests laughter releases pleasure hormones, reduces stress, and functions as an analgesic (Jones & Tanay, 2016).

Humor has also been recognized to serve multiple social functions: It can alleviate tension (Sen, 2012), facilitate discussion of painful topics (Browne, 2016; Chapple & Ziebland, 2004; Schöpf, Martin, & Keating, 2017), foster solidarity (Holmes, 2000; Meyer, 2000; Robinson, 2009), allow speakers to acknowledge, and cope with, the complexities of social reality (Chapple & Ziebland, 2004; Fox, 1990), and challenge prevailing power dynamics (Davidson, 2001). Previous health literature has identified these facets of humor as valuable, particularly within the context of clinical and research relationships (Chapple & Ziebland, 2004; Jones & Tanay, 2016). Indeed, a number of analysts have argued that humor can help facilitate difficult interactions regarding health and illness (Robinson, 2009; Schöpf et al., 2017). For instance, in their exploration of how men talk about testicular cancer, Chapple and Ziebland (2004) posit that “patients might use jokes to introduce ‘awkward’ topics and to convey messages about taboo subjects, such as death, that might be unacceptable if conveyed seriously” (p. 1225). However, vanishingly little has been written about how humor might be utilized in discussions of *health policy*. This distinction is important insofar as the relationships individuals develop with their bodies and their clinicians are tied up with, but nonetheless distinct from, the relationships they develop with health policy and policy-actors, broadly construed (e.g., academics, politicians, activists). Furthermore, the substantive issues and power dynamics raised by discussing health on an individual scale often differ from those raised when discussing issues salient to health policy (e.g., who is deemed to possess “expertise,” within and outside the research space)—as such, they require separate consideration. In response to this research lacuna, this article seeks to extend discussions of health research and humor beyond the personal and toward the political—fully acknowledging the mutually constitutive nature of each.

Accordingly, this article seeks to highlight times at which humor performed the social functionalities identified above, within the context of citizens’ juries discussing health policy. In so doing, it pays particular regard to questions of power and its negotiation. It also, simultaneously, reflects on humor’s darker side—its ability to deride, divide, and ostracize. Acknowledging the normative duality of humor, we explore the ethical questions which arise when we consider its use as a “facilitative tool” in group research encounters. As extant health literature has identified, concerns regarding the ethical implications of humor—and by extension its appropriateness in professional (e.g., clinical) relationships (see Jones & Tanay, 2016)—are plentiful. For example, according to superiority theory, humor is necessarily cruel and therefore ethically suspect (Watson, 2015). However, this position is premised on the belief that *all* humor originates from a sense of superiority, which has, as discussed, been widely criticized (Morreall, 1989). Others have expressed concern that botched humor runs the risk of belittling its intended recipients, insofar as failing to “get” a joke may render some “affronted, baffled and taken in rather than amused” (Watson, 2015, p. 412). More broadly, some have expressed fear that humor’s inherent absurdity makes it unfit for inclusion in serious conversation, for example, discussions of mortality and morbidity (Morreall, 1989). We contend, however, that it is possible to frame humor as a social phenomenon which cannot only avoid ethical pitfalls but also satisfy some ethical responsibilities. For instance, some health theorists have emphasized humor’s capacity to inject fun and amusement into clinical spaces (Jones & Tanay, 2016), and we are inclined to frame this as a dimension of humor which can assist in the creation of ethical health research spaces. We would argue, in essence, that it is ethically questionable to ask participants to spend protracted periods of time engaged in group research, without creating space for fun and levity. Furthermore, we would suggest that the simultaneously positive and negative potential of humor—its ability to, for example, signify solidarity through shared knowledge or division through derision—calls for a nuanced analytical approach to its use in research. Yet, while (health) researchers have paid significant attention to humor as both a broad social phenomenon and a reality of group research encounters (Browne, 2016; Janhonen, 2017; Robinson, 2009), only a limited literature considers humor as a research practice (see Grønnerød, 2004, for a notable exception).

In addition, while numerous health analysts have addressed the role of humor within the less lengthy format of focus group research (see Browne, 2016; Davidson, 2001; Janhonen, 2017), their work tends to focus on participant production of humor, paying limited regard to the role facilitators play. One exception recommends that research facilitators hold open a space “for lightness, laughter and humor” (Browne, 2016, p. 203) but provides limited guidance as to how this might be achieved. More commonly, existing literature focuses on the production of humor by participants of group-based research (often involving marginalized communities) and considers how researchers should respond (Davidson, 2001), with several authors recounting a reluctance to make any active contribution to humorous exchanges. Illustratively, in reflecting on her research with smoking mothers, Robinson (2009) relates her decision to refrain from introducing or coproducing humor while facilitating focus groups. She explains that although she sporadically felt inclined to smile at something intended as humorous, she “never respond(ed) with a joke . . . never introduc(ed) a joke . . . and distanc(ed) [herself] from the actual production of humor” (p. 274). She did this, she explains, in deference to her role, positionality, and power relative to her participants, as well as her responsibilities in responding to, and representing, women’s lives and their smoking. This observation raises important questions regarding the potential of humor as a “facilitative tool,” prompting us to ask, “How might we use humor ethically, particularly when discussing serious matters and/or in contexts characterized by potential power imbalances?” While these questions have been addressed with regard to *clinical* relationships (see Jones & Tanay, 2016), little has been done to consider the matter in terms of *health research* relationships, particularly those developed during policy-oriented (rather than medically or microsocially oriented) research.

Beyond this, there appears to be an even greater paucity of work exploring the role of humor within deliberative research encounters which are, as intimated, increasingly popular within health research. To illustrate, while Kashefi and Mort (2004) briefly allude to deploying humor as a facilitative tool in jury activities, they do not reflect on why or how they did so or to what effect. This is significant because, as discussed, citizens’ juries and focus groups differ: The former tends to last for more protracted periods, and facilitators often play a more directive role. Furthermore, while the methodology of focus groups can be used with a view to redressing the prevailing power dynamics of knowledge production (Davidson, 2001), citizens’ juries pursue this aim by design and therefore necessarily demand attention to performances of power—something which humor can help to mediate.

## Power

In this article, we conceive of power as a fluid, situational, and hybrid force mediated by immediate and ingrained social relationalities, rather than as straightforwardly inscribed in, or possessed by, particular individuals (Smith, 2006). Specifically, we reject the idea that research relationships are (or should be) characterized by constant and stable asymmetries in power—positioning the researcher (the subject of study) in a steady hierarchical position above the researched (the object of study) (Ben-Ari & Enosh, 2013). Instead, we view power as a relational, oscillating force mediated by multiple factors which are negotiated and exercised in a variety of ways, by a variety of people, across a variety of contexts (including the evolving topics of discussion within a research setting). Accordingly, the orientation and functionality of power should be inductively identified, rather than deductively assumed. In keeping with this perspective, this article demonstrates how humor was used within our citizens’ juries to (temporally) obtain, relinquish, exercise, and destabilize power within the research space. We primarily focus on the negotiation of power through humor as a dynamic arising within the research process, though we occasionally seek to highlight how inextricably linked aspects of researcher and participant positionality *more broadly* (e.g., gender) came to bear on localized practices of power. Finally, we seek to highlight how participants deployed humor to respond to power asymmetries located outside of the research space, a concern which is particularly salient in health policy research.

## Method and Analysis

Our three 2-day citizens’ juries were undertaken in July 2016 in Glasgow (20 participants), Liverpool (20 participants), and Manchester (17 participants). These cities were purposively sampled, on the basis that they all have large health gaps within their populations. They also share a similar sociopolitical context, including the shared experience of post-industrial decline. Taken together, these factors have led to previous comparative studies of health inequalities across the three cities (e.g., Walsh, Bendel, Jones, & Hanlon, 2010). We commissioned Ipsos MORI to recruit participants in each city, using a mixture of door-to-door and in-street approaches. Recruiters were provided with a target profile for the 20 recruits with the aim of ensuring the sample was representative of the U.K. population in relevant sociodemographic and attitudinal terms (quotas were applied for gender, age, social class, working status, and political views, as well as attitudes toward public health). Recruitment was undertaken in the two weeks leading up to each jury. Recruiters worked on weekdays and weekends, during the day and in the evening, and across areas of each city to maximize the chances of making contact with a broad range of people. Recruiters were asked not to select people with preexisting relationships to one another or who had taken part in a discussion event or focus group in the last year. The profile of recruits was broadly in line with the quota targets, notwithstanding a slight overrepresentation of SNP voters in Glasgow, and Green party voters in Manchester, in comparison with the current voting profiles of these cities.

The research was approved by the University of Edinburgh’s Ethics Committee on July 2, 2016. The recruiters provided all potential participants with information sheets to explain the project, as well as consent forms to consider in advance. The researchers then further explained the consent forms during the first session of each jury. Having had an opportunity to ask questions, all participants were asked to sign the consent forms (it was explained, verbally and in writing, that participants were free to leave at any point, whether or not they signed the consent forms, and could withdraw consent at any point during the research). All participants opted to sign the consent forms, which are stored in a secure cabinet at the University of Edinburgh, and none subsequently withdrew consent.

During each jury, participants undertook a range of exercises to get to know each other, to develop and agree with their own “rules of engagement,” and to find out more about health inequalities research and proposed policy solutions. This included hearing from two “witnesses” in person and four via prerecorded, specially commissioned videos (which can be viewed here: http://www.healthinequalities.net/understanding-health-inequalities). A range of experts acted as “witnesses,” including academics, public health campaigners, and a general practitioner (clinician). Each provided a different perspective on health inequalities and potential policy responses, with the intention of reflecting current research and policy perspectives in the United Kingdom. Jurors were given an opportunity to develop questions in small group discussions and then to reconvene as a full group, at which time they could put their questions directly to the “witness” or to facilitators with health inequalities research expertise. The program for each day also included several social breaks. There were therefore multiple opportunities for jurors to hear from each other and express themselves. Each jury culminated in a collective voting exercise, during which jurors voted on potential policy responses to health inequalities.

Across the 2 days, we collected data in three ways: (a) individually, via questionnaires which we asked participants to complete at the beginning of the juries, at a midpoint, and at the end, and collectively, via (b) ethnographic notes throughout (including during social breaks) and (c) audio recordings of all full and small group discussions (multiple recording devices were utilized to capture the latter). This article draws exclusively on data generated via the second and third methods. The research team consisted of six members who took on the following roles: facilitator (consistently Oliver Escobar); ethnographer (consistently Gillian Fergie); small-group facilitators (Oliver Escobar, Rosie Anderson and Rebecca Hewer); critical friend, to help jurors critically consider and challenge the evidence and views they were encountering (Katherine Smith, assisted by Sarah Hill); questionnaire lead (Alex Wright); data recorder (Alex Wright); time-keeper (Alex Wright); and photographer (Alex Wright). Finally, jurors were asked to undertake a range of “sticky wall” exercises which involved writing suggestions on pieces of paper which were then displayed on a sticky wall and, in some cases, voting on preferences. Jurors received £220 following participation, in recognition of the significant time commitment.

Transcriptions of audio recordings were imported into NVivo 10 and initially coded by K.S., following the abductive development of a thematic coding framework. This involved K.S. constructing an initial set of codes, which related to current research and policy debates on health inequalities and to some of the questions we asked jurors to consider, before adding and refining codes while coding three (of the 45) transcripts. This initial coding was checked by R.H., who then coded the remaining transcripts, during which time she further refined and added codes. The fully coded data were then re-read in full by K.S. (the initial coder), at which point a small number of text sections were attached to codes but no further codes were added. The code “humor” was, initially, inductively generated in recognition of the fact that numerous respondents explicitly identified “having a laugh” as something they valued. Thereafter, both explicit discussions of humor and instances which R.H. recognized as implicitly humorous were coded. Subsequently, “humor” was identified as a compelling dimension of jury discussions, warranting further exploration. At this juncture, notable trends and patterns, explicated in what follows, were observed.

Where possible, sections of the transcribed data coded as “humor” were further explored in the ethnographic field notes. This was done by triangulating information about the timing and substantive content of events, with contextual information contained in the notes, which were tagged with timings related to the progress and structure of discussions. This allowed for exploration of any observed nonverbal acts, visual cues, or gestures, and therefore enhanced our understandings of seemingly humorous interactions or statements. As a fundamentally dialogic achievement, recognizing humor relies on knowledge of how a joke was both intended and received (Chapple & Ziebland, 2004; Holmes, 2000). In this article, intent and reception are primarily inferred by the analysts according to their understanding of humor theory and common linguistic structures, as well as their lived experience of the citizens’ juries. The use of ethnographic data works to redress any limitations created via this analytical approach.

## Findings: Reflecting on the Social Functionality of Humor in Our Citizens’ Juries

During our citizens juries—and in deference to the principles of deliberative research (Wakeford, 2002), critical research theory (Karnieli-Miller, Strier, & Pessach, 2009; Mies, 1993), and our critical understanding of power (above)—we sought to challenge the subject/object divide assumed to prevail, and nurtured, within more positivist approaches to qualitative research. We sought to recognize potential power asymmetries conferred by our immediate and relatively ingrained positionalities and to behave in ways which challenged hierarchical or stratified arrangements (Davidson, 2001). We were cognizant, for instance, that participants might view us as “health (policy) experts” (and indeed, at times, we represented ourselves as having particular kinds of expertise) and that our symbolic relational positioning as “subject to their object” might create a sense of inequality. Furthermore, we were cognizant that social relations relating to ethnicity and social class might permeate interactions with a subset of jurors (given we were a predominantly White/middle-class research group and had striven to recruit demographically diverse participants). In response, we deployed a number of equalizing tactics. We held the juries in spaces we felt were relatively public (a Glasgow concert hall in a main shopping area, Manchester’s central library, and a Friends’ Meeting House in Liverpool); we wore casual clothing rather than “office work” clothes (in an effort to present ourselves as friendly and accessible and diminish any outward signifier of difference); we ate alongside participants at lunchtime and engaged in informal (nonjury-related) discussions where appropriate; and we consciously endeavored to engage with participants in a jovial fashion. The intent was to be friendly, approachable, and involved, rather than engaged in the maintenance of clear boundaries or power asymmetries.

Following the conclusion of the juries and the inductive exploration of data, we became increasingly cognizant of how frequently—albeit prereflexively—we utilized humor to achieve these ends. Closer analysis of this emergent finding suggested that humor played a significant role in how researchers and participants negotiated, exercised, and renounced power to create a more equal and amicable research environment. In addition, it became apparent that humor enables particular forms of negotiation with evidence of health inequalities and arguments regarding health policy. In deference to this, in what follows, we highlight researcher uses of humor, collaboratively generated humor, and participant uses of humor, as well as humor as a lubricant and humor as an abrasive. Throughout, we highlight the social functionality of humor, particularly in regard to discussions of health policy, as well as the general negotiation of power.

## Negotiating Power: Researcher Uses of Humor

When meeting jurors for the first time, the principal facilitator, O.E.—whose continental origins are rendered undeniable by his Spanish accent—would introduce himself in the following way:

I’m Oliver, and as you can tell by my accent I am from south [pause] Glasgow.

This joke often elicited disproportionately loud laughter. Here, relief theory may prove illuminating. Tensions born of uncertainty about, and unfamiliarity with, the research process were pleasurably deflated and released as O.E. confounded possible apprehension among the jurors (Meyer, 2000). The incongruity in O.E.’s joke was twofold—it confounded expectations with regard to his origins *and* the potential character conferred by his position as a researcher (e.g., a serious man). Furthermore, O.E.’s joke was—if not self-deprecatory—oriented toward the self. This served two social functions. First, it ensured no juror would feel demeaned by the introduction of humor. It was obvious that O.E. was not expressing contempt or hostility toward anyone but himself (if that)—no one but O.E. could feel laughed *at*. On reflection, self-oriented humor can be marked relatively “safe”—as a genre of humor with minimal social risk. Second, in destabilizing the potential perception that O.E. occupied a position of superiority or power, the joke began the work of challenging the object/subject relationship which can pervade more hierarchical research encounters. As Meyer (2000) and Holmes (2000) observe, in inviting those who appear to occupy a less powerful position than we do, to laugh at some aspect of ourselves, we begin to equalize relationships. Balancing power relations, in this way, may be considered particularly salient to health research generally, where researchers may be presumed to have not only expertise in the focus of the research but also medical training or a professional role within a healthcare setting (Hewitt, 2007).

Building on this, R.A. and R.H. used parody—a style of humor which relies on a kind of outlandish mimicry of its object (Hirsch, Kett, & Trefil, 2002)—in ways which arguably worked to disrupt power asymmetries. Both women performed caricatures of the symbolic positions they putatively inhabited (researcher/facilitator), exposing their largely unstable and limited character. In Liverpool, R.A. suggested she might withhold food from her participants:

Participant: Oh we’re running into lunch.

R.A.: No, there’ll be no lunch for you!

While in Manchester, R.H. made light of the research team’s relative dependence on respondent choices—as they selected which public health policy measure they would recommend:

R.H.: This is the last vote, you can’t mess it up. [Everything] will just crumble down, I’ll be fired, Alex will be fired, Rosie will be fired, and it’ll be all your fault.

Participant: Oh it’s a big responsibility! I reckon I’ll be OK.

Here, via “parodic performances” (Nentwich, Ozbilgin, & Tatli, 2015, p. 239) of difference in positionality and power, we played with the symbolic separation of researcher and researched. Holmes (2000) identifies two differing functionalities of this type of humor-based power play: introducing informality (renouncing power) and masking coercion (exercising power). To illustrate, our parodies could be understood as democratizing attempts to inject levity and informality into relationships potentially characterized by hierarchy and to subsequently eschew the potential possession of power. As Butler (2006) famously observes, parody can trouble prevailing power dynamics. Alternatively, R.A. and R.H. could be understood as disguising essentially coercive interactions by using humor to sugar the pill of a directive. Perhaps R.H. wished to ensure completion of the questionnaire, and R.A. the continuation of her conversation without an immediate break for food. So read, R.A. and R.H.’s parodic performances would work to sustain, rather than challenge, power asymmetries—allowing both to “do power” without being viewed as authoritarian. These dual—somewhat contradictory—interpretations point to the paradoxical nature of humor, to the importance of phenomenological readings (e.g., intent and receipt), and to the complexity of power play. Reflecting on this particular paradox, we are inclined to suggest—in contrast to Holmes, who appears to portray the “sugared directive” as necessarily repressive—that these two ways of doing power might not be mutually exclusive. A parody can be more easily dismissed than a straightforward instruction—laughed away, perhaps—and the introduction of humor might license participant uses of levity to contest facilitator power (as we discuss later). Thus, a sugared directive may, in fact, invite an equalizing informality which provides a less authoritarian and more democratic way of getting things done (a necessity within the tightly structured method of citizen’s juries). Here, then, the duality of humor might be better understood as the coexistence of putatively contradictory functionalities within one interaction, rather than the production of one of two distinct outcomes.

An exploration of the humorous dimension of these verbal interactions reveals parody to be slightly riskier than self-oriented joking. Our intent was that our jesting be encoded as “so patently absurd as to cast doubt on the overt claims of what [was] being characterised” (Suto, 1979, cited by Watson, 2015, p. 413). There was a danger, however, that participants might interpret our statements as genuinely intended and be “taken in.” Read accordingly, both excerpts appear ethically questionable. Our own reading, however—augmented by reference to ethnographic notes and participant evaluation data—is that the jurors interpreted our parodies as parodies. After seemingly being denied food, R.A.’s group of participants continued their conversation unperturbed, whereas the coproduction of humor in R.H.’s encounter speaks to a level of jovial complicity on the part of the juror with whom she was speaking. The apparent success of these encounters may be better understood by situating them within their immediate social and temporal context. Both took place on the second day of proceedings, once relationships had been built and rapport achieved. Their “safety” as humorous interludes was at least partially dependent on a preestablished context of lightness and shared understanding. As intimated, humor is differentiated as such by reference to the context of its delivery (Davidson, 2001; Janhonen, 2017; Lippitt, 1994). Our parodic statements were identifiable as incongruous at least partially because they followed shared experiences which contradicted them.

## Across the Object/Subject Divide: Researcher and Participant Coproduction of Humor

Facilitator efforts to create an egalitarian space (e.g., equalize power asymmetries) were often aided by jurors’ uses of humor to build solidarity. Solidarity can be produced through humor in multiple ways. Laughter is performative and interactional and can be used to signal acceptance, forming social bonds (Robinson, 2009). Furthermore, because much humor relies on a shared understanding of social logic and its disruption, parties to a joke must be members of a common knowledge community (Browne, 2016; Davidson, 2001; Fox, 1990). Relatedly, solidarity can be fostered—as Holmes (2000) suggests—through “interactively achieved [and] jointly constructed” (p. 165) jokes. Take the following:

P1: Even the rats now are like super rats because of the fast food.

P2: Because of the fast food yeah.

P1: Giant killer rats in Liverpool.

P2: Honestly it’s overrun with rats in Liverpool.

P1: They’ve got super rats.

R.A.: They don’t tell you that when you get off the train.

P1: [The advice on eating five fresh fruits and vegetables a day is] so simple, over simplified. Apparently it was also, before it was government policy in the 90s it was made up by people who sold fruit and veg.

R.H.: I’m not sure about that, but we can see about clarification, the fruit and veg mafia.

P1: That’s it.

P2: Greengrocers revolt.

Here, facilitators and jurors coproduced humor—the former eschewing the role of observer (as adopted by Davidson, 2001; Robinson, 2009) and creating collaborative jokes with the latter. This worked to build a sense of shared purpose among researchers and the researched, framing our research as a joint enterprise and the research space as more equal. This instance also demonstrates an arguably successful subversion of the notion that the researchers were absolute experts in health policy matters, insofar as participants claimed expertise and contributed insights.

## Negotiating Power: Participant Use of Humor

Beyond this, participants frequently used humor to trouble and disrupt potential power dynamics, without facilitator assistance. Sometimes, they did so by using humor as a critical and oppositional discourse. For instance, when asked to reflect on what she would take away from the first day of deliberations (which had included some discussions of health-damaging behaviors, but which had also tried to move beyond these behaviors to consider more “upstream” causes of health inequalities), one participant joked,

Well its healthy food, healthy lifestyle, don’t smoke, don’t drink, don’t breathe, and don’t eat.

Some participants also joked with each other during a break, in response to the first evidence session:

Animated discussion in the break space related to the issue of characterising outcomes for rich and poor. “They make it sound like if you are poor you are set for the knacker’s yard.” (Field notes)

Humor is naturally subversive: It disrupts dominant meaning (Fox, 1990; Mulkay, 1988) and can be used to critique and undermine prevailing genres of discourse (Watson, 2015). It is therefore primed to contest and resist hegemony and its proponents. Used in a citizens’ jury, this resistance can perform power in two ways. First, it can trouble dominant forms of knowledge (e.g., hegemonic discourses) understood to shape available evidence or influence how jurors evaluate policy proposals. In Liverpool, the satirical^2^ representation of behavioral responses to health inequalities can be read as a critical engagement with individualizing discourses, and in Manchester, the juror’s aside can be viewed as a means of critiquing narratives perceived as stigmatizing. This particular function of humor seems especially likely to arise in health *policy* research, given that political frames are, arguably, more likely to be denaturalized via parodic engagement than their medical counterparts. Medical discourses are routinely reified via appeals to science (Gilbert, 2008), whereas political narratives are often publicly debated. Indeed, in asking jurors to critically engage with policy solutions, we invited them to reject, disrupt, and trouble them. Second, when directed at those believed to promote these discourses, humorous comments can facilitate conflict by softening critique and are therefore particularly apposite modes of address for those seeking to criticize someone perceived to be more powerful. Humor therefore appears to provide participants with a way to claim and exert some power, disrupting asymmetries produced through the perpetuation of hegemonic discourse or positionality. Reflecting on this, and its positive implications, we suggest it may pay dividends to be affirmatively responsive to participants’ humorous contributions, as—indeed—we were.

This recommendation comes with the caveat that participants and individual researchers, themselves, should not *generally* become the punch line to a critical joke. There is something of a distinction (albeit an unstable one) between satirizing an opinion and satirizing the person who utters it—one we would be keen to observe. Indeed, while we may wish to support humor which works to trouble the object/subject divide, our understanding of power as situated, fluid, and susceptible to being troubled suggests participants may obtain, and use, power against researchers in ways which are potentially problematic. While humor was not used in this way within our research encounters, there were other interactions which were arguably marked by problematic (gendered) power dynamics. Indeed, our data reflect instances in which female researchers were apparently condescended to by male participants or framed as the male facilitator’s “staff” (rather than as colleagues/experts). Here, then our more socially ingrained positionalities mediated our relative power (Rose, 1997). Clearly, health research takes place within a complex web of relationships which intersect to dilute or intensify power and its performance.

The socially meaningful characteristics which arguably influenced our positionality within the research space—and our subsequent relationships to citizen jurors—did *occasionally* arise as an issue within uses of humor by participants. However, it arose when such social characteristics could be understood to redouble the power conferred by researcher positionality. To illustrate, O.E.’s intersectional identity of *male* researcher—which had the potential to intensify power asymmetries with female participants—occasionally became the focus for subversive contestation. For instance, two participating women—one in Glasgow and one in Liverpool—jokingly professed an attraction to O.E. One such interaction went as follows:

O.E.: One voice at a time please, I need to be strict because we need to finish on time.

Participant: Yes, I like it when you’re strict.

O.E.: You’re going to make me blush.

While drawing largely on O.E.’s position as principle facilitator, the humor in this comic interaction was also reliant on prevailing gender and age dynamics. It was O.E.’s position as a powerful *man* which made the above romantic advance appear socially acceptable and humorous, rather than inappropriate and troubling. Through the subversion of gender ideologies—the rejection of female desire as submissive and inactive—the participant offered another resistant “parodic performance.” This performance was made all the more parodic, as the participant in question was visibly older and therefore confounded the cultural desexualization of women in late adulthood. That said, some queer theorists warn against overestimating the subversive potentiality of this kind of humor, arguing that by relying on and highlighting difference, gender parodies can work to re-ingrain the heterosexual matrix (Butler, 2006; Nentwich et al., 2015). This points to a possible limitation of humor as a tool for subversion within the research space, and indeed more broadly. In humor’s dependence on the subversion of social logic, it holds the potential to reify that logic and further polarize power asymmetries. Again, the paradox of humor is its duality, about which we should remain reflexive.

Power, or lack thereof, was also mocked in more straightforward ways—particularly kinds of power without immediate embodiment in the research space. Several participants made fun of politicians: One juror suggested their local government “couldn’t run a bath,” whereas another parodied processes of political change:

We’ll sort [corporate taxes] out: we’re going down to Westminster after this, me and you, on two horses. You’re a Spaniard, you must know how to ride a horse, and we’re going down there to deal with this. [directed to O.E.—facilitator—in a full group discussion]

As intimated previously, policy research involves the development and consideration of a range of relationships not necessarily salient within more individual approaches to the exploration of health. Here, the participant demonstrates how humor can assist in the representation and negotiation of these relationships, discursively. In addition, beyond parodying the process of achieving political change, this humorous excerpt, like so many already explored, is layered with meaning and functionality. It creates solidarity by drawing on a shared cultural knowledge of fairy tales and the Wild West, white knights, and sheriff cowboys. Furthermore, it relies on in-group membership, in so far as two of the figures of fun, included in the narrative, were well-known figures to the group but not necessarily beyond. The joke is funnier, in short, if you are able to imagine the participant and O.E. riding to Westminster on horses. There are multiple levels on which to “get” the joke—and the ability to “get” it largely depends on, and ingrains, in-group identity. It is also illuminating to consider the substance of this joke, something we discuss in the following section.

## Difficult Discussions: Humor as a Coping Mechanism

A sense of political powerlessness pervaded all of the citizens’ juries. Indeed, a number of participants expressly reported feeling there was vanishingly little they could do to challenge the seeming ubiquity of health inequalities. Political powerlessness is likely related to, but distinct from, a feeling of powerlessness over one’s health or in one’s health relationship and is thus a substantive topic peculiar to health policy research. To illustrate, this particular feeling appeared to be compounded by a prevailing view that political actors were inefficient, uncaring, and untrustworthy. Ethnographic notes taken in Manchester reflect this:

Video plays. Discussion in pairs. Two female participants get animated, one pulls out her chair conveying her disgust at [evidence suggesting] politicians are not addressing preventable deaths, then listens intently to her partner. [. . . ] She expresses frustration at the messages conveyed in the evidence.

On occasion, these sentiments were communicated so strongly that we, as the research team, felt compelled to reflect on the ethics of drawing attention to the prevalence of health inequalities when participants had limited political power to redress them. Here, something of a paradox was created, for—as Ben-Ari and Enosh (2013) remark—participant access to experiential knowledge gives them power to shape and influence research encounters and the data generated. Here, the felt powerlessness of the participants exercised a particular power over the research team.

In any event, the above participant’s joke was arguably an attempt to invite levity into a subject sadly bereft of joy, to render the felt experience of powerlessness less powerful. This is a routinely recognized function of humor in health research (Chapple & Ziebland, 2004; Jones & Tanay, 2016; Rose, Spencer, & Rausch, 2013; Schöpf et al., 2017). Indeed, it has been suggested that humor can work not only to inject a lightness into sad or awkward topics but also to facilitate their discussion. For instance, Chapple and Ziebland (2004) demonstrate how men suffering from testicular cancer use “tumour humor” to allow for familial and peer explorations of their sensitive health experiences. Our data adds to this body of work, insofar as it demonstrates that participants often sought to use humor to deal with discussions of population health inequalities, rather than personal experiences of illness. That the topic of population health is arguably less experientially salient is notable: It is curious that it prompted similar coping methods. In any event, these findings suggest that humor was more than a derivative of our research encounters or merely a “facilitative research tool” which enabled certain interactions—rather it was a generative force allowing for the production of data (Robinson, 2009).

This observation introduces an ethical consideration previously raised by both Davidson (2001) and Grønnerød (2004): How should we respond to humorous interludes underpinned by serious subtexts? Both within the research space and, later, in our analytical efforts? If participants speak with levity about issues of gravity, should we respond with lightness or seriousness? This is an area of particular concern for health researchers, given the frequency with which serious and potentially distressing topics are explored within the field. Discussions of morbidity, mortality, and well-being are always potentially fraught. There are no easy answers and we should confess that we (the researchers involved) had not considered this issue in advance. Looking over the data suggests that the relatively lengthy duration of the citizens’ juries allowed us to respond with a multiplicity of tones and discursive styles, across the 2 days of deliberation, in a way which allowed us to reintroduce topics of gravity previously dismissed through levity. While we created spaces for levity, we also facilitated serious discussions about ill health, bereavement, and prevailing inequalities. That said, if we were to conduct similar research again, this is one area we would aim to collectively and reflexively consider before meeting with jurors.

## Negotiating Power: Humor as a Social Lubricant

Participants not only used humor to navigate power with researchers and political actors but also their relationships with each other. Humor was routinely used as a “social lubricant” (Meyer, 2000, p. 317)—a way to cope with potentially fraught interactions and ease tensions which might otherwise arise or fester. To illustrate, in Glasgow, a dispute about the relative value of statistical evidence over personal anecdote simmered between some of the jurors over the 2 days of deliberation. Indeed, ethnographic data suggest these debates continued into participant break-times. In one of the final sessions of the juries, one participant—who favored the use of statistical evidence—offered this:

I’m… going to commit the rest of my life to finding a cure for [other participant’s] cigarette smoking friend [who] is the only statistic that matters when it comes to smoking.

To which the intended recipient knowingly responded,

You can’t be 100%, and I’m unanimous on that.

This interaction could be interpreted as combative, an attempt to exert power through sarcasm, and defiant resistance—but delivery, tone, reception, and atmosphere implied unification. A form of closure was achieved: An ongoing dispute was settled with a joke (Grønnerød, 2004). Here, again, context was key: What came before ensured jovial receipt of that which might otherwise be construed as insulting—in sum, the jurors were familiar with one another. As Holmes (2000) observes, “insults between those who know each other well are also signals of solidarity and markers of in-group membership” (174). Furthermore, one could argue that the deliberative character of the research encounter licensed disagreement and provided a space within which this kind of humorous interaction was both enabled and enabling—authorized by the acceptance of discord, allowing for discussion of that discord. Jokes of this nature may have appeared less apposite in a space otherwise characterized by consensus and harmony.

## The Darker Side of Humor: Derisive Power Plays and Seeming Spite

Other uses of humor to navigate power within participant relationships were more ambivalent. For instance, in Liverpool, a participant who made the repeated suggestion that policy proposals be categorized by reference to a series of largely inscrutable acronyms was teased by a fellow participant:

P1: And also PIP is a social benefit now in this country, taking over the disability. So it’s kind of a good acronym.

P2: And the other good thing is no one would know what it meant.

This interaction was captured by the recording, and consequently the transcription, but was not witnessed by the facilitators—it is difficult, then, to analyze its normative dynamics. It is possible this teasing was good-natured, an artifact of friendship. It is similarly possible that the teasing participant sought to express genuine displeasure regarding the opacity of acronyms, and deliver a sugared directive, using humor as a vehicle to do so. As Holmes (2000) observes, humor can “license” the expression of negativity otherwise deemed inapposite. Interpreted as such, this joke might better be understood as a social abrasive, rather than a lubricant—a divisive tool apt to pry people apart, bringing people down (hierarchically) rather than together (Meyer, 2000). Indeed, the use of humor to communicate controversial meaning has been identified as coercive: A joke is more difficult to contest than a sincere utterance (Holmes, 2000; Robinson, 2009). Coercion is often identified as a key vessel for the expression and exercise of power (Ben-Ari & Enosh, 2013). This is perhaps best exemplified by the following interaction between another participant and a facilitator:

P: You’ve literally cut me off twice then.

F: No, I haven’t cut you off, it’s just . . . .

P: I was joking about that.

Here, a participant frames a complaint as a joke, thus preventing the facilitator from properly addressing their grievance. Displeasure is expressed and no recourse allowed.

On very rare occasions, participant uses of humor *appeared* more hostile. In one city, we encountered a participant who seemed to struggle to integrate with fellow jurors. This struggle manifested in his verbal interruptions—which were read as tangentially related to the topic of group conversation—and expressions of frustration when it was felt these interruptions were side-lined. Furthermore, ethnographic notes reflect that, unlike most other participants who left their belongings unattended when on break, this participant kept possession of personal belongings, perhaps indicating lack of trust in jurors and facilitators. Toward the end of the second day, some participants began audibly laughing at this juror. Our immediate response, as facilitators, was to openly treat the struggling juror with as much compassion as we could—to model tolerance and understanding—and to avoid any engagement with the hostile laughter. On reflection, we feel this tactic was inadequate. Of course, it does not automatically follow that by being open to positive manifestations of humor we created a space for hostility to flourish. One could argue that by modeling positive forms of humor, and by otherwise nurturing an amicable space, we reduced the likelihood of contemptuous laughter. We are inclined to conclude, however, that we might have responded more effectively had we critically reflected on possible uses of humor in our preparation for the juries. While the risks inherent to humor may never be entirely overcome, they can certainly be reduced. For example, had we discussed this kind of possibility in advance, we would have been better prepared and might have raised the issue with the jurors involved, as early as possible, inviting them to reflect on how the ways they used humor related to the earlier rules of engagement they had themselves helped develop and agree.

What we might also conclude is that productive or happy participation in lengthy deliberative fora is aided by the possession of certain social competencies, including the capacity to encode, appreciate, and produce humor. For while humor may trouble potential distributions of power, it does so by conferring a type of power on the joker and therefore a kind of social privilege. Those incapable of, or uncomfortable with, engaging with humor may therefore find themselves at a social disadvantage, particularly in spaces where humor is encouraged. Again, this points to the duality of humor—for some it can provide a facilitative tool with which to play with power, but for others it can present as yet another privilege they simply do not have access to. This is a particularly potent observation for analysts who might wish to deploy humor to address uneven power typographies within group research encounters. For health researchers generally, these issues are particularly pertinent, especially where topics of group discussions can be stigmatizing to individuals and where participants may bring varied social competencies. For health *policy* researchers, they demand a particular attention to how individuals engage and cope with political debate and discord.

## Conclusion

We opened this article by highlighting the dearth of literature reflecting on uses of humor by participants *and researchers* in group-based research settings. We noted that this was a particularly notable gap with regard to deliberative spaces, like citizens’ juries, which differ from more commonly used group-based methods in health research, such as focus groups, in both their prolonged duration and their implicit aim of troubling hierarchical distributions of power. We also noted that while the role of humor in discussions of personal health had been attended to in existing health literature, little had been said about how humor might function in health *policy* research. By reflecting on three citizens’ juries focusing on policy responses to health inequalities, we have argued that cautiously inviting humor into these spaces allows for the ongoing negotiation of power via the disruption of traditional research relationships, the creation of rapport and solidarity, subversion and resistance, the abatement of anxieties, and the facilitation of difficult discussions. Although we had not (as we now feel we should have), given any serious collective consideration to the potential role of humor prior to conducting the juries, the data explored here highlight how we, as facilitators, used humor (alongside casual dress and informal conversation) as a “facilitative tool” throughout, helping us to destabilize the power asymmetries often sustained by traditional research relationships. In turn, by producing and coproducing humor, we arguably held open a space for lightness, enabling jurors to similarly produce and use humor in positive ways.

Generally, the space we held open was occupied by participants in ways which nurtured positive outcomes. Jurors deployed humor to negotiate power dynamics, cultivate relationships, build solidarity, and explore difficult dimensions of social reality. Furthermore, humor allowed for the articulation of insights which might otherwise have gone unsaid—controversial, provocative, resistant articulations. There were, however, fleeting instances when humor appeared to play a less positive role—laughter seemed derisive or jokes manifested as thinly veiled criticisms. It was at these junctures that we were encouraged to truly confront the duality of humor: its multivalent qualities and potential ethical pitfalls. With these insights in hand, it is possible to interpret many facilitator uses of humor, within the research space, as largely prereflexive negotiations of rhetorical risk—privileging self-oriented joking and parody over ridicule and derision.

In drawing these explorations together, we have concluded that humor should be used, and facilitated, reflexively and critically in this kind of prolonged research encounter. Furthermore, we have posited that humor poses particular opportunities for health *policy* researchers. This is meaningful, as qualitative investigations of population-level health issues and policies—which address unfair differences in health outcomes by social group—move beyond individual accounts of experience. Indeed, while our accounts of humor were in some ways similar to those described in broader health literature—particularly with regard to discussions of difficult topics—they were also distinct in their concern for local communities and the role of elite decision makers in affecting health experiences. In sum, our focus on the health inequalities experienced by local communities, and the role of policy decisions within this, led jury discussions to shift between personal and abstract discussions. We suggest these kinds of health policy–focused discussions pose a range of particular challenges, including the negotiation of contestable knowledge, feelings of political powerlessness, and resistance against ostensibly (rather than opaquely) structural forces. We have suggested that the additional challenges of working to explore such complex health policy topics can be met, in part, by employing appropriate methodological tools and engendering considered researcher reflection—particularly on rapport-building through the sensitive use of humor.

Finally, we suggest that humor should be deployed with an appreciation of its contextual and contingent character. “Safe” humor is a dialogic achievement which draws from a shared understanding of what is apposite, incongruous, absurd, and well-meant. When (co)produced in a deliberative research space, humor can be facilitative, illuminating, and equalizing. Of course, the degree to which we can proactively put our reflexive insights into practice—exert control over the use of humor, invite it or prevent it from taking place—is debatable. What we would suggest, however, is that by holding a space open for lightness, modeling gentle joviality, engaging in the co-construction of jokes, and responding positively toward (nonderisive) participant uses of humor, our research team increased the likelihood of humor being used more broadly and more positively.

In our future research endeavors we intend to proactively reflect on how we might use humor, and how humor might emerge, in group-based health research encounters. We believe such reflexivity may work to reduce any feelings of uncertainty and trepidation regarding the role of humor in research and increase our ability to use it in an ethical and facilitative manner. In addition, we believe such reflexivity will render us better equipped to respond to instances in which humor appears to be impacting negatively on any participants. We would urge others to do the same.

## Notes

1. When it appeared to make instances of humor intelligible.

2. Satire is a style of humor which “mocks social conventions, another work of art, or anything its author thinks ridiculous” (Hirsch, Kett, & Trefil, 2002)
